# Digital Twinning of Interorgan Communications

**DOI:** 10.1002/cph4.70002

**Published:** 2025-02-09

**Authors:** Lance Fortnow

**Affiliations:** ^1^ Illinois Institute of Technology Chicago Illinois USA

## Abstract

Recent advances in our ability to collect and process information, particularly through artificial intelligence, opens up some exciting possibilities for understanding interorgan communication and treating conditions that arise from that communication breaking down. We describe a vision of a digital twin of interorgan communication that will give us a testbed for virtually researching, teaching and searching treatments, greatly increasing our capabilities to understand and manage the complex interactions in our bodies.

## 1 Introduction

Imagine an intelligence that, connected to the organs in the human body, coordinates the organs so that they work well together ensuring that each organ receives what it needs to function properly and each organ provides what the other organs need to work well. Even when directly not in the communication path of the organs, this intelligence has provided a system that allows them to communicate directly with each other. When something goes wrong, the intelligence finds ways to try to correct the problem or the intelligence can communicate with health professionals to describe symptoms that are causing the problems.

Of course, we already have such an intelligence called the human brain. To quote the editor‐in‐chief (Raj 2025), the brain acts like a conductor coordinating the organs to play a beautiful symphony we call a well‐working human body, either directly from signals sent to and from the brain and the other organs, or indirectly as organs communicate directly with each other.

If we already have such a great natural intelligence, what role can artificial intelligence play in interorgan communication? Quite a bit, especially as the body and brain battles aging and disease.

Communication is defined as the transmission or exchange of information. Information in the body gets transferred in many different mediums, including neurological signals, hormones, chemicals, proteins, amino acids, migrasomes and many others including some that we likely have yet to discover. Information gets transferred through a variety of different channels from the nervous system, the bloodstream, molecules that transfer to neighboring cells, and physical stretching of tissue, among many others.

Interorgan communication connects the various parts of our bodies in an extremely complex system. The human body might be the most complex system on this planet. Artificial Intelligence has shown a remarkable ability in recent years to help tame complexity, think of natural language translation or protein‐folding predictions. Likewise if we wish to understand the ways our body communicates, we must take advantage of these new tools and techniques.

Artificial intelligence thrives on data, and the information that transits through the human body is a treasure chest that can reveal how the body functions, what might go wrong and how to mitigate the damage. To best apply AI, we need a way to model and capture this information, ideally a digital twin of interorgan communication that allows us to research, study and treat diseases in a safe digital environment.

### 1.1 Digital Twinning

Digital twinning is digital simulation of a physical process. For example, you might make a digital twin of a car, a factory or a building before it is even built and be able to run simulations to create data, test various scenarios and predict using artificial intelligence to ensure that you create the right car, factory or building. We can do something similar with various functions of the human body including interorgan communications.

Consider an Amazon warehouse. Amazon maintains a completely digital twin of the very physical items in the warehouse (NVIDIA 2023), knowing every item's location and how it moves in, out and through carefully handling deliveries, the movement of goods through automation, robots and human workers, managing incoming orders, packaging and shipping the goods out of the warehouse. Amazon uses AI extensively to optimize item location, time to move through the facility, robot usage, packaging, and recovery from any disruption. Amazon not only has a digital twin of each facility but its whole distribution network, ensuring that your toaster gets to you quickly.

Imagine if we could do the same for our bodies, creating a digital simulation of our bodies (Attaran and Attaran 2024). Interorgan communication is a great place to start, because AI thrives on information, and interorgan communication is just the transference of information throughout our bodies.

We could create a twin for a healthy person, a person with a specific ailment, or even for a specific individual. Once we had the twin, we could use statistics and machine learning to make sense of the complex interactions of the communications among organs and try to map functionality from the messages that get sent. We could introduce diseases into the digital twin and run simulations to see the effects. We could introduce artificial aging to see how the communication process might break down. We could introduce digital treatments, whether it be pharmaceuticals, surgery or implants, to see how it might mitigate diseases and aging. We can do this all without having to perform potentially dangerous experiments on physical humans.

We would have to carefully test any treatment, at least as carefully we test potential new drugs. But the capability to generate and test potential treatments in a digital world will greatly speed up our ability to fix our bodies when they don't work properly.

## 2 The Digital Twin

Below we discuss various aspects of digital twinning of interorgan communications, first looking at what we can do with a digital twin, how to build it, how to collect the needed data and last, but not least, the ethical considerations that we must consider through this whole process.

### 2.1 Applications of a Digital Twin

Once we create a digital twin, we open up a wealth of applications that would have been difficult, time‐consuming, expensive or impossible if we had to do experiments solely on animals and humans. A digital twin is a platform that allows many applications. We'll talk about a few below but if history is any guide, the best applications of a digital twin are those we haven't even thought of yet.

Primarily a digital twin would provide an incredible opportunity for research as we can study and analyze data on all aspects of interorgan communication. In the digital twin, we can disrupt communication mechanisms and see how that contributes to conditions like systemic inflammation, sepsis, or autoimmune diseases. We can simulate the progression of conditions such as chronic kidney disease and its impact on other organs, and investigate diseases involving multiple systems such as when COVID‐19 affected the lungs, hearts and kidneys (Wang et al. 2023).

We can test drugs and other treatments for effectiveness and safety in a virtual environment before, but not instead of, animal and human trials. Researchers can analyze the effects of combined multiple therapies on interconnected organs and predict organ‐specific or systemic toxicities, especially for drugs targeting multiple pathways. We can automate trials, exploring many treatments at once.

We can simulate aging (Kirkwood 2005) and how it affects interorgan communication, such as the impact of reduced kidney function on cardiovascular health, or impaired neural activity and transmission.

Epidemiologists can use the digital twin as part of a larger system to model the spread of diseases across populations and the effect of large‐scale interventions such as vaccines or isolation.

Digital twins can provide strong individualized treatment as well. We can try to create a digital twin that simulates a specific human, constantly updated with data from wearables, such as blood glucose monitors and ECG devices, to help with diagnosis and decision‐making. Doctors can Identify early warning signs or biomarkers for disease onset by simulating how diseases such as diabetes or cardiovascular disorders develop based on the individual's data and test how the patient may respond to specific drugs and doses by simulating drug metabolism and organ‐level effect and test potential side effects, especially for multisystem conditions. We can also model the impact of surgical procedure on interconnected organs.

We can model how a donated organ will integrate throughout a patient's system and simulate immune system interactions to anticipate organ rejection. Doctors can also simulate how a patient will respond to prosthetic devices, implants, artificial organs and neural stimulators.

Doctors can use visualization, data and simulations to help patients understand how different behaviors, such as diet, exercise and medication regimens, can impact their health.

Finally, students can use digital twins to learn about interorgan communication and understand how to diagnose various rare diseases in the virtual environment, similar to the way pilots learn to handle rare challenges in an airplane simulator.

### 2.2 Creating the Digital Twin

Creating a digital twin of interorgan communication would require a major collaboration of computer scientists, biomedical engineers, medical professionals and research scientists including physiologists, biochemists and biophysicists. The development of a digital twin follows a traditional data science life cycle (Loukides and Patil 2011): Defining the problem, collecting data, cleaning and preparing the data, training an AI model that would lead to the digital twin, evaluating the model and continuously refining the model, often with additional data that starts the cycle all over again. Finally, we use the model as we described in the previous section.

First, we'd have to set the scope of the project, which organs, biological processes and communication channels to include. To gain the full power of a digital twin we should try to include as many organs, processes and channels as possible as the interaction between them drives the power of these organs to work well together. For practicality, one should pick individual communication processes, like hormones released by endocrine glands into the bloodstream or signals derived from gut microbiota, and create simulations of those processes and then later on start putting them together.

We can also incorporate data from in vitro and in vivo systems of increasing complexity including multi‐cell‐cultures, organoids, and (multi‐)organs‐on‐a‐chip models. While these systems provide controlled environments for testing hypotheses and validating model predictions, we need to be careful about their limitations when training models for human communication. The processes in these simpler systems may not fully capture the complexity of human biology.

We need to capture rich accurate data from multiple sources, including clinical data, biomarkers to track hormones and cytokines, physiological measurements, medical imaging, RNA sequencing, behavior data such as diet, exercise and stress issues and continuous data from wearables. We'll go into details of the type and methods for data collection below. We'd have to do this data collection for a large number of people, from various demographics and levels of health. Biomedical and electrical engineers can play a critical role here, helping to develop safe, non‐invasive and cost‐effective methods to collect the data we need to create an effective digital twin.

Cleaning and preparing the data can be the most time intensive part of the process. We need to make sure that we've properly collected the data, properly connected the data from various sensors together and put the data in the right format for the next step of model training. This would be a great place to bring in students at various degree levels, train them and put them to work on this process to give them the experience in connecting artificial intelligence with medical research.

We then need to choose the right machine‐learning model for training. Given that communication depends on timing, most likely we'd use the transformer model (Vaswani et al. 2017) that has proven so powerful for human language communications, such as translation and large‐language models. Such training would require extensive computing power and access to specialized chips, such as high‐level GPUs, through a partner's computing network or via the cloud. After training, we need to validate the model using additional data.

At each stage, we should work with the research scientists in physiology, biochemistry and biophysics to help us find deficiencies in the model and suggest new measurements and data that can help us build more robust models. Hopefully, this interaction becomes two‐way, opening up potential new understandings of communication that scientists could explore further. We should be careful though not to overuse our previous knowledge in the training of the model since learning tends to work better when we don't give it any constraints.

Transformer models usually create opaque black‐box algorithms; we cannot understand how they work other than by their input/output behavior. There is a growing area of “Explainable AI” that aims to better explain their behavior, perhaps leading to new insights into physiological and pathophysiological regulatory processes. However, explainable AI may dramatically limit the model's effectiveness to the point that we lose more than we gain.

We then have our simulation of interorgan communication, at least for some form of communication. We can run simulations and test out various scientific hypotheses, or even use AI to help suggest some hypotheses themselves. We will continuously improve the model with more data, and by integrating data from different communication methods and channels. Ideally, we could finetune the model, to capture interorgan communication when some disease prevents ideal circumstances, or fine tune to have a simulation of a specific individual.

None of these steps would be easy. It would take a couple of years to produce a usable digital twin for a single communication channel, and possibly decades to develop a digital twin for most interorgan communication. Advances in artificial intelligence and medical device technology might shorten these time frames. The hardest organ to simulate would be the human brain. But, new techniques in artificial intelligence give us a fighting chance, at least for communication.

### 2.3 Data Collection

The variety of interorgan communication and the information that goes through our bodies, sometimes at the molecular or electron level, will make getting good information for training difficult and expensive, at least initially until we develop new technologies, perhaps partnering with biomedical engineers. At every stage of the process, we also must make the privacy and accuracy of the data paramount.

Wearables (Shajari et al. 2023) provide the simplest continuous way to gather data. The public already uses devices like smart watches, rings and glucose monitors regularly and we can add specialized devices to pick up specific biomarkers. But not everyone can afford or wishes to use wearables, and the information they provide gives us limited insight into interorgan communication.

We can use imaging technologies such as MRI, PET Scans and Microscopy to obtain snapshots of organ‐level interactions, metabolic activity and high‐resolution visualizations of cellular and molecular interactions. But, these can only capture some of the activity and not over a long time period. We should also collect data on genetic sequences and perform detailed blood tests to measure proteins involved in inflammation, such as interleukins, tumor necrosis factors, and interferons. These proteins help organs communicate with one another, especially during immune responses.

We can use molecular biomarkers to track hormones, cytokines, and neurotransmitters in blood or other bodily fluids to map endocrine and paracrine signaling pathways. Ultracentrifugation, size‐exclusion chromatography, and immunoaffinity capture are used to isolate extracellular vesicles like exosomes and microvesicles. We can use molecular cargo analysis to characterize RNA, proteins, and lipids carried by extracellular vesicles.

Neural activity might be the most difficult to capture (Buzsáki et al. 2012) but perhaps the most important method the brain uses to communicate with the organs. For now, we need to use electrodes placed on or into the brain and other organs to capture the electrical activity of the brain (EEG) and the muscles (EMG). Hopefully, advances in technology and AI analysis of data can lead to less invasive and safer methods.

This is only a sampling of the various ways we can try to collect information on patients. Hopefully this digital twinning project and others like it will accelerate research on new technologies that allow us to collect interorgan communication in ways that let us get a larger picture in a safe cost‐effective manner.

### 2.4 Ethical Considerations

A digital twin of human systems, such as interorgan communication, raises a host of ethical challenges that don't manifest themselves in, say, a digital twin of an Amazon warehouse (Bruynseels et al. 2018).

Most importantly, we must ensure the privacy of individual data, through the data collection process and especially if we create a digital twin of a specific person to meet HIPAA requirements and patient concerns. Even a general digital twin trained on data collected from several people could potentially reveal the training data of a single individual if not handled properly (Dwork and Roth 2014). Systems must have multiple layers of security to limit access of the data to appropriate individuals and limited access that prevent revealing individualized information to those who should not have access to it.

Patients must give informed consent for using the information collected from them and understand the benefits and risks of gathering that information particularly if it uses internal monitors.

We need to ensure data are collected from a broad demographic spectrum to avoid bias and inequity (U.S. Food and Drug Administration 2020). We must also ensure that the methods of collection itself are free from biases, from the devices themselves to those doing the collecting. Digital simulations, particularly of an individual, could be quite expensive and we need to find ways for everyone who can benefit to have the opportunity to do so.

The digital twins should be used to help humans but all technologies can also be misused accidentally or on purpose and safeguards must be put in place to limit abuse. In the worst case, a terrorist could use digital twins to simulate the effects of a biological weapon to maximize its effects so we need to be careful who has access and what they can do with the digital twin.

We need a proper regulatory framework for digital twins of interorgan communication and our bodies in general, balancing the ethical needs with the need for innovations that may improve and save lives.

Everyone needs to understand that the simulations of a digital twin may not always correspond to what happens in an actual human body. Particularly any treatment must be tested in a clinical environment as we do today. Digital twins help us accelerate the process of finding treatments but they don't replace testing them in the real world.

Managing these concerns might seem overwhelming, but not creating digital twins will lead to many suffering from diseases we could have treated or mitigated. Lack of trust in digital models can also have negative outcomes so we need to train our doctors when to trust the data more than intuition.

## 3 Conclusion

Recent huge advances in data analytics and artificial intelligence opens up the possibilities of digital twins of interorgan communication. It will take time, care, resources and new technologies to truly realize this dream, but the promise of a digital system that allows us to provide better care and new treatments for disease and aging will dramatically advance the importance of interorgan communication for the betterment of the human experience.

## Conflicts of Interest

The author declares no conflicts of interest.

## Data Availability

Data sharing is not applicable to this article as no new data were created or analyzed in this study.
